# Pathophysiological changes in the cerebellum and brain stem in a rabbit model after superior petrosal vein sacrifice

**DOI:** 10.1042/BSR20171043

**Published:** 2018-11-21

**Authors:** Lei Cheng, Pin Guo, Yi-wei Liao, Hong-liang Zhang, Huan-ting Li, Xianrui Yuan

**Affiliations:** 1 Department of Neurosurgery, The Affiliated Hospital of Qingdao University, Qingdao, Shandong 266003, China; 2 Department of Neurosurgery, Xiangya Hospital, Central South University, Changsha, Hunan 410008, China

**Keywords:** brain stem, cerebellum, edema, K+, Na+, SPV

## Abstract

In certain surgical procedures, sacrificing the superior petrosal vein (SPV) is required. Previous studies have reported transient cerebellar edema, venous infarction, or hemorrhage that might occur after sectioning of the SPV. The present study investigated the pathophysiological changes in cerebellum and brain stem after SPV sacrifice. Rabbits were divided into the operation group where the SPV was sacrificed and the control group where the SPV remained intact. Each group was further subdivided into 4, 8, 12, 24, 48, and 72 h groups which represented the time period from sacrificing of the SPV to killing of the rabbits. The water content (WC), Na^+^ content, K^+^ content, and pathophysiological changes in cerebellum and brain stem tissue were measured. In comparison with the control, the WC and Na^+^ content of cerebellar tissue were increased in the 4, 8, 12, and 24 h operation subgroups (*P*<0.05), but only increased in the 4-h subgroup of the brain stem tissue (*P*<0.05). The K^+^ content of the cerebellar tissue decreased in the 4, 8, 12, and 24 h operation subgroups (*P*<0.05) but only decreased in the 4-h subgroup of brain stem tissue (*P*<0.05). Nissl staining and TEM demonstrated that cerebellar edema occurred in the 4, 8, 12, and 24 h operation subgroups but not in the 48- and 72-h subgroups. Brain stem edema occurred in the 4-h operation subgroup. In summary, cerebellum and brain stem edema can be observed at different time points after sacrificing of the SPV in the rabbit model.

## Introduction

The superior petrosal vein (SPV), identified by Dandy [1], is located in the cerebellopontine angle near the rostral aspect of the trigeminal nerve. The danger of damaging or sacrificing the pestrosal vein and its branches should be considered during surgical procedures [2].

In certain circumstances, safe sacrificing of the SPV should be performed, for example sometimes the SPV should be sacrificed due to its close proximity to tumors [3]. The SPV is also usually exposed during posterior fossa surgery [4]. It has been reported that this vein can be sacrificed without complication [5]. However, some previous studies have reported that transient cerebellar edema [6] and venous infarction [7], brain stem infarction [8], or even hemorrhage might occur after sacrificing of the SPV [9]. For instance, Samii et al. reported that amongst 30 patients who underwent sacrifice of the SPV, 9 patients had severe complications, of these 7 patients had cerebral edema and 1 patient had venous cerebral infarction [4]. Masuoka et al. [6] reported the case of a 77-year-old female patient who underwent microvascular decompression for the treatment of trigeminal neuralgia and the SPV was sacrificed in order to obtain a good surgical field. On postoperative day 1, the patient developed a headache and nausea followed by a decreased level of consciousness. MRI revealed an extensive venous infarction in the right cerebellum [6].

Recent studies on the SPV were almost always focussed on autopsy and clinical observation, while a few studies used an animal model to look at venous infarction and other complications after the SPV was sacrificed. In the present study, a rabbit animal model was used to observe the pathophysiological changes in the cerebellum and brain stem after sacrificing of the SPV.

## Materials and methods

### Animals and groups

#### Animals

All animal studies conformed to the National Institutes of Health Guide for the Care and Use of Laboratory Animals (NIH publication number 85-23, revised 1996). The protocol was approved by the Ethics Committee of Xiangya Hospital, Central South University.

#### Groups

One hundred forty four New Zealand white adult male rabbits were provided by the Experimental Animal Center of Xiangya Hospital, Central South University. These clean grade rabbits were 6 months old with an average weight of 1.81 ± 0.14 kg. All rabbits were divided into two groups including an operation group (the SPV of the rabbits were sacrificed) and a control group (the rabbits underwent same procedure as the operation group except the SPV remained intact). Each group was subdivided into six subgroups which included 4, 8, 12, 24 48, and 72 h subgroups according to the time when the rabbits were killed after the procedure, and each subgroup had 12 rabbits. According to the different methods for treating the brain tissue, each subgroup was further divided in half (group A and group B), each group containing six rabbits. Both A and B contained six rabbits respectively. The rabbits in group A were killed to study the water, sodium, and potassium content in the cerebellum and brain stem, and the rabbits in group B were killed to perform histopathological assessment of cerebellum and brain stem.

### Surgical procedure

The rabbits were anesthetized using 3% sodium pentobarbital (China Pharmaceutical Shanghai Chemical Reagent Company, Shanghai, China) and then were fixed on the operating table in a prone position (the external occipital protuberance at the highest point). The hair of the rabbits in the operative field was shaved. Medical alcohol was used to disinfect the incision and the sterile towels were used to cover it. A straight incision in the skin was carried out along the midline. The incision began approximately 1 cm cranial from the external occipital protuberance and extended to the craniocervical junction. The muscles were split from the occipital bone. A homemade retractor was used to pull aside the skin and muscles. The occipital bone was ground under the microscope to form the bone window. The boundary of the bone window was defined as follows: there was approximately 1 cm from the external border to the midline (exposed edge of the sigmoid sinus); the inner border was slightly farther than the center line; the rostral border showed the inferior edge of the sigmoid sinus; there was 0.7 cm from the caudal border to external occipital protuberance. The dura was cut in the shape of a cross (+) to expose the petrosal vein. The SPV was cut after it was coagulated with the bipolar, and then the SPV was cut. Gentamicin saline (160 U/ml) was used to wash the operative cavity before closing the dura to make sure no bleeding occurred. The dura was restored to its original position and covered by gelatin sponge. The muscle and skin were sutured stratified using surgical thread. The rabbits in the control group were operated using the same procedure except sacrifice of the petrosal vein did not occur. Further details were provided in a previous paper [10].

### Water content measurement

Rabbits in group A were killed at different time points, and then the cerebellum and brain stem taken out within 5 min. The weight of the cerebellum and brain stem were measured within 5 min from the start of the brain removal procedure. The dry weight was measured after the cerebellum and brain stem were placed in 104–110°C electric heating oven (Changsha Instrument Factory, Changsha, China) for 24 h. In order to ensure complete water removal, the measurement was recorded after a constant weight was obtained. The water content (WC) of the cerebellum and brain stem were calculated according to the Elliott formula.

### Na^+^, K^+^ content measurement

Establishment of a sodium standard curve: the serial sodium standard solutions (20, 40, 60, 80, 100 mmol/l) were prepared using sodium standard solution obtained from Beijing Chemical Works, Beijing, China. The extinction value (A) of each standard solution was measured using an XY402 atomic absorption flame spectrophotometer (Shenyang Analytical Instrument Factory, Shenyang, China) at 589 nm. The linear regression equation of the extinction value and sodium concentration (C) was then calculated. The linear range of sodium was 20–100 mmol/l.

Establishment of a potassium standard curve: the serial potassium standard solutions (20, 50, 80, 110, 140 mmol/l) were prepared with potassium standard solution (Beijing Chemical Works, Beijing, China), and then the extinction value (A) was measured using the XY402 atomic absorption flame spectrophotometer (Shenyang Analytical Instrument Factory, Shenyang, China) at 766.5 nm. The linear regression equation of the extinction value and potassium concentration (C) was then calculated. The linear range of potassium was 20–140 mmol/l.

Measurement of Na+ and K+ content: the cerebellum and brain stem samples which had been used to determine the dry weight were placed in 1 ml concentrated nitric acid overnight in order to start the digestion of the samples. The samples were then digested with 2 ml of concentrated perchloric acid until without the production of white smoke. Then 3% concentrated nitric acid containing 3% strontianite (SrCO_3_) was used to dissolve the samples to 5 ml. Na^+^ and K^+^ extinction values were measured with a spectrophotometer at different wavelengths.

### Histopathological assessment of cerebellum and brain stem

Rabbits in groups B were anesthetized with pentobarbital at the designed time points and perfused transcardially with saline followed by 4% paraformaldehyde (Tianjin Chemical Reagent Research Institute, Tianjin, China). The cerebellum and brain stem were taken out and stored overnight in a 100 mM phosphate buffer (pH =7.4) containing 30% sucrose. They were subjected to Nissl staining and electron microscopic observation using an H-7500 TEM (Hitachi, Japan).

### Statistical analysis

All data were expressed as mean ± S.D. Statistical analyses were performed using the SPSS 22.0 software (SPSS, IBM, IL, U.S.A.). *P*<0.05 was considered statistically significant.

## Results

### WC of cerebellum and brain stem increased in the rabbit model with the sacrificed SPV

**Figure 1:**
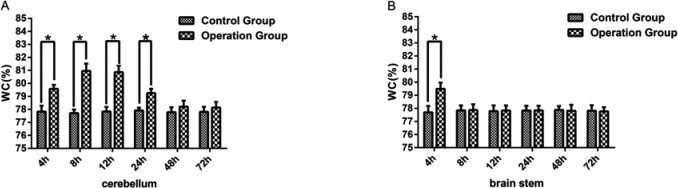
WC was increased in the cerebella and brain stem (**A**) Represents the WC level of cerebella in the 4, 8, 12, 24, 48, 72 h subgroup of the operation group and the control group. (**B**) Represents the WC level of the brain stem in the 4, 8, 12, 24, 48, 72 h subgroups of the operation group and control group. The SPV was sacrificed in the operation group but remained intact in the control group. The data were presented as mean ± S.D., *n*=6. * represent *P*<0.05 compared with the control subgroups.

To begin with, in the present study, a comparison of WC amongst the 4, 8, 12, 24, 48, and 72 h six control subgroups was performed using cerebellar tissue. The results showed that there was no significant difference in WC amongst the different subgroups of the control group (*P*>0.05). Then the WC of the operation subgroups was determined and compared with the appropriate control subgroup. Compared with the control group, there was a significant increase in the WC of the 4, 8, 12, 24 h subgroups of the operation group (*P*<0.05) (Figure 1A). However, no significant difference was found between the 48 and 72 h subgroups of the operation group (*P*>0.05) (Figure 1A). The difference in WC between subgroups of the operation cohort was also studied. The results showed that amongst the operation subgroups WC in the 4, 24, 48, 72 h subgroups was significantly different compared with the results of 8 and 12 h subgroups (*P*<0.05) (Table 1).

**Table 1:** Comparison of WC of the cerebellum amongst the six operation subgroups

Subgroups	WC (%)	*P*-value (compared with 8-h subgroup)	*P*-value (compared with 8-h subgroup)
4 h	79.58 ± 0.31	*P*<0.05	*P*<0.05
8 h	80.96 ± 0.56	*P*>0.05	*P*>0.05
12 h	80.87 ± 0.48	*P*>0.05	*P*>0.05
24 h	79.25 ± 0.34	*P*<0.05	*P*<0.05
48 h	78.21 ± 0.46	*P*<0.05	*P*<0.05
72 h	78.14 ± 0.43	*P*<0.05	*P*<0.05

The WC (%) in the operation group was represented as mean ± S.D. *P*<0.05 was considered statistically significant.

In brain stem tissue, WC was also investigated amongst the six subgroups of the control group and there was no significant difference in WC amongst the different control subgroups (*P*>0.05). The WC between the control group and operation group at each subgroup time point was compared. There was a significant increase in the WC in 4-h operation subgroup compared with the control group (*P*<0.05) (Figure 1B). However, no significant difference was found in the 8, 12, 24, 48, and 72 h subgroups of the operation group compared with the control group (*P*>0.05) (Figure 1B).

### Na^+^ content in cerebellum and brain stem increased in the rabbit model with the sacrificed SPV

**Figure 2:**
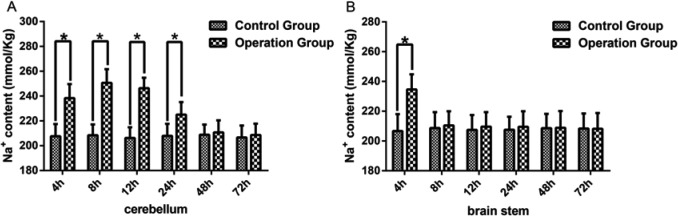
Na^+^ content was increased in the cerebella and brain stem (**A**) Represents the Na^+^ content level of cerebella in the 4, 8, 12, 24, 48, 72 h subgroup of the operation group and control group. (**B**) Represents the Na^+^ content level of the brain stem in the 4, 8, 12, 24, 48, 72 h subgroups of the operation group and control group. The data were presented as mean ± S.D., *n*=6. * represent *P*<0.05 compared with the control subgroups.

Sodium ions play a major role in maintaining osmotic pressure. To further explore the impact of SPV unilateral disconnection, the change in Na^+^ content was measured in the cerebellum and brain stem using the rabbit model both in the operation group and the control group. In cerebellar tissue, the Na^+^ content was compared amongst the six control subgroups, and the results showed that there was no significant difference in Na^+^ content amongst the different subgroups (*P*>0.05). Then the Na^+^ content was compared between the control group and operation group for each time point. There was a significant increase in the Na^+^ content in the 4, 8, 12, and 24 h subgroups of the operation group compared with the control group (*P*<0.05) (Figure 2A). There was, however, no difference between the operation group and control group in the 48 and 72 h subgroups (*P*>0.05) (Figure 2A). The difference in Na^+^ content between subgroups of the operation cohort was then also studied. Then the Na^+^ content differences in the operation group were performed through comparing the 4, 12, 24, 48, and 72 h subgroups with 8-h subgroup and comparing 4, 8, 24, 48, 72 subgroups with 12-h subgroup. The results showed that amongst the subgroups of the operation group, Na^+^ content in 4, 24, 48, and 72 h subgroup was significantly different compared with the results of 8 and 12 h subgroups (*P*<0.05) (Table 2).

**Table 2:** Comparison of Na^+^ content of the cerebellum amongst the six operation subgroups

Subgroups	Na^+^ content (%)	*P*-value (compared with 8-h subgroup)	*P*-value (compared with 12-h subgroup)
4 h	238.36 ± 11.34	*P*<0.05	*P*<0.05
8 h	250.65 ± 10.98	*P*>0.05	*P*>0.05
12 h	246.32 ± 8.47	*P*>0.05	*P*>0.05
24 h	225.05 ± 10.11	*P*<0.05	*P*<0.05
48 h	210.74 ± 9.69	*P*<0.05	*P*<0.05
72 h	208.63 ± 9.10	*P*<0.05	*P*<0.05

The Na^+^ content (%) in the operation group was represented as mean ± S.D. *P*<0.05 was considered statistically significant.

In the brain stem, Na^+^ content was also compared amongst the six control subgroups, and between the control group and operation group at each subgroup time point. The results showed that no significant differences in Na^+^ content amongst the different subgroups of the control group were found (*P*>0.05). Compared with the 4-h control group, there was a significant increase in the Na^+^ content in the 4-h subgroup of the operation group (*P*<0.05), while no difference was observed in the 8, 12, 24, 48, and 72 h subgroup of the operation group compared with that of the control group (*P*>0.05) (Figure 2B).

### K^+^ content in cerebellum and brain stem decreased in the rabbit model with the sacrificed SPV

**Figure 3:**
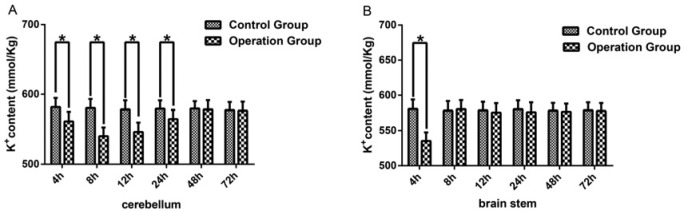
K^+^ content was increased in cerebella and brain stem (**A**) Represents the K^+^ content level of cerebella in the 4, 8, 12, 24, 48, 72 h subgroup of the operation group and control group. (**B**) Represents the K^+^ content level of the brain stem in 4, 8, 12, 24, 48, 72 h subgroup of the operation group and control group. The data were presented as mean ± S.D., *n*=6. * represent *P*<0.05 compared with the subgroups of control group.

As potassium ions also play an important role in maintaining osmotic pressure, the K^+^ content was also measured using the unilateral disconnection of the SPV rabbit model. In cerebellar tissue, the K^+^ content amongst the 4, 8, 12, 24, 48, and 72 h control subgroups was compared, and no significant difference was found in K^+^ content amongst the different subgroups (*P*>0.05). The K^+^ content was then compared between the control group and operation group for each subgroup. The results showed that there was a significant decrease in the K^+^ content in the 4, 8, 12, and 24 h operation subgroups compared with the corresponding control subgroup (*P*<0.05) (Figure 3A), no difference was found in the 48 and 72 h subgroups (*P*>0.05) (Figure 3A). Then the K^+^ content differences between each operation subgroup were compared. The 4, 12, 24, 48, and 72 h subgroups were compared with 8-h subgroup and the 4, 8, 24, 48, 72 h subgroups with 12-h subgroup. Amongst the operation subgroups K^+^ content in the 4, 24, 48, and 72 h subgroup was significantly different from the 8 and 12 h subgroups (*P*<0.05) (Table 3).

**Table 3:** Comparison of K^+^ content of the cerebellum amongst the six operation subgroups

Subgroups	K^+^ content (%)	*P*-value (compared with 8-h subgroup)	*P*-value (compared with 8-h subgroup)
4 h	561.46 ± 13.73	*P*<0.05	*P*<0.05
8 h	540.18 ± 12.65	*P*>0.05	*P*>0.05
12 h	546.32 ± 13.51	*P*>0.05	*P*>0.05
24 h	564.82 ± 12.97	*P*<0.05	*P*<0.05
48 h	578.37 ± 13.13	*P*<0.05	*P*<0.05
72 h	576.55 ± 12.82	*P*<0.05	*P*<0.05

The K^+^ content (%) in the operation group was represented as mean ± S.D. *P*<0.05 was considered statistically significant.

In brain stem tissue, the K^+^ content amongst the six subgroups of the control group was also compared. Results showed that there was no significant difference in K^+^ content amongst the different subgroups in the control group (*P*>0.05). The comparison of K^+^ content between the control group and operation group for each corresponding subgroup was performed. Compared with the corresponding control group, there was a significant decrease in the K^+^ content in the 4-h operation subgroup (*P*<0.05) (Figure 3B). There was no difference between the operation group and the control group for the 8, 12, 24, 48, and 72 h subgroups (*P*>0.05) (Figure 3B).

### Pathological changes in the cerebellum and brain stem in the rabbit model with the sacrificed SPV

#### Nissl staining

To study the pathological changes in the cerebellum and brain stem in SPV unilateral disconnection rabbit model, Nissl staining of cerebellum and brain stem was performed on all subgroups of both the operation group and the control group.

**Figure 4:**
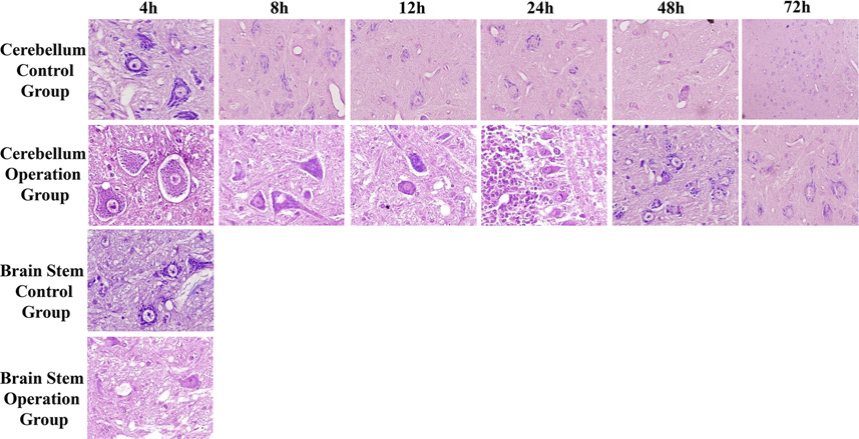
Nissl staining results for cerebellum and brain stem in the 4, 8, 12, 24, 48, and 72 h subgroup of both the operation and control groups The top row shows Nissl staining results of the 4, 8, 12, 24, 48, and 72 h subgroup in cerebellum control group. The second row shows Nissl staining results of the corresponding operation subgroup. The third and fourth rows show Nissl staining results of the 4-h subgroup in brain stem operation group and control group respectively.

The cells in the cerebellum tissue in each control subgroup had an intact cell structure (Figure 4A–F), and were arranged neatly. The staining of the cytoplasm was clear. Nissl bodies in these cells were rich, coarse, and plaque-like. In contrast with the 4-h operation subgroup, the neurones were swollen and arranged irregularly. The cell structure was also blurred. Plaques of Nissl bodies in neurones became smaller (Figure 4G). In the 8-h subgroup of the operation group, the cell changes were similar to the results of 4-h subgroup, while only a small number of Nissl bodies were seen in the neurones (Figure 4H). In the 12 and 24 h operation subgroups, the neurones were also swollen and arranged irregularly. In the 24-h subgroup, the Nissl body disappeared in some cells (Figure 4I,J). The plaques in Nissl bodies could be observed in the 48-h subgroup, and the swelling of cells was alleviated (Figure 4K). In the 72-h subgroup, the nuclei of cells were slightly stained and the cell structure was normal (Figure 4L). In the brain stem in the 4-h operation subgroup, Nissl bodies disappeared in some cells and the cells were arranged irregularly (Figure 4N). The cell structure in the 4-h control subgroup was normal and Nissl bodies could be observed (Figure 4M). No obvious abnormality was found in the other subgroups of the operation group.

#### TEM results

**Figure 5:**
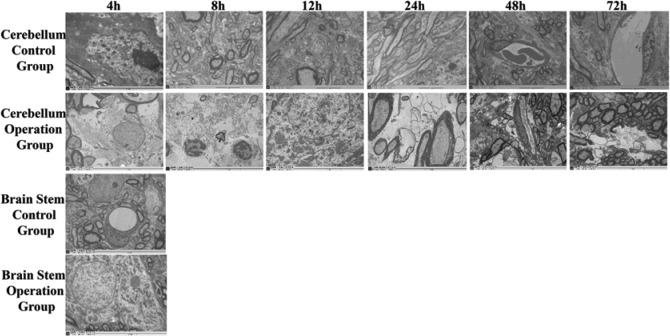
TEM results for cerebellum and brain stem in the 4, 8, 12, 24, 48, and 72 h subgroup of operation and control group The top row shows TEM results of the 4, 8, 12, 24, 48, and 72 h subgroup in the cerebellum control group. The second row shows TEM results of the corresponding operation subgroups. The third and fourth rows show TEM results of the 4-h subgroup in the brain stem operation group and control group, respectively.

The ultrastructure of the cerebellum in the control group was normal. The nerve cells and glial cells were not swollen, and mitochondria and endoplasmic reticulum could be observed clearly and, the nuclear membrane was clear and recognizable (Figure 5A–F). After 4 h of sacrificing of the SPV, the neurones and glial cells were swollen; the structure of mitochondria was blurred and vacuole-like changes in mitochondria were observed and, some endoplasmic reticula were slightly dilated (Figure 5G). In the 8 and 12 h operation subgroups, most neurones and glial cells were swollen and vacuoles appeared in the cytoplasm. The structure of mitochondria was blurred. The endoplasmic reticulum was dilated and the nuclear membrane unclear. The apoptosis of neurones was obvious, with the nuclear condensation and chromatin margination. Local necrosis of neurones and inflammatory cell infiltration were also observed. Edema was obvious around the capillaries (Figure 5H,I). The changes in the ultrastructure of the 24-h subgroup were smaller than that of the 8 and 12 h subgroups (Figure 5J). The structure of the mitochondria and endoplasmic reticulum was clear. Degeneration of mitochondria in both the axon and neurone was observed. In the 48 and 72 h subgroups, the pathological changes of the cerebellum were small (Figure 5K,L). The structures of most neurones were normal except some nerve fibers demonstrated edema.

There was no obvious abnormality in the ultrastructure of the brain stem in the control group (Figure 5M). Mild structural changes in neurones and nerve fiber edema were observed in the 4-h subgroup of the operation group (Figure 5N). No obvious abnormality was found in the other operation subgroups.

## Discussion

It is common for a neurosurgeon to decide whether the SPV can be safely sacrificed when it limits the exposure of surgical targets. Although it has been reported that SPV can be sectioned without complication, unfavorable outcomes may occur after dividing the SPV [5,9,11,12]. A recent clinical study revealed that the sacrificing of the SPV during surgery was associated with a complication rate that is significant in frequency and severity compared with leaving the vein intact [13]. Sacrificing of the SPV may lead to serious and potentially life-threatening complications. Previous studies of the SPV were focussed mainly on autopsy and clinical observation. A large number of animal experiments and clinical studies have been carried out on arterial obstruction but only a few studies had been performed on venous infarction and other complications using animal models. In the present study, the same rabbit model described previously to observe the pathophysiological changes of the cerebellum and brain stem after sacrificing of the SPV was used [10].

The Na^+^ and K^+^ content of the cerebellum and brain stem was examined initially. It was found that in the group in which the SPV was sacrificed, the water and Na^+^ content of cerebellum and brain stem were increased, while the K^+^ content decreased. The change of Na^+^/K^+^ content may be explained by the activity change of sodium/potassium-ATPase (Na^+^/K^+^-ATPase). In mammalian cells, the basic role of Na^+^/K^+^-ATPase is to maintain the balance of ion gradients between the intracellular and extracellular environment. The ion gradients play an important role in maintaining normal cell volume and normal morphology of the cell membrane. ATP is required for keeping the ion balance between the inside and outside of cells. Under normal physiological condition, ATP-dependent ion pumps (such as Na^+^/K^+^-ATPase) can consume approximately 50% of the ATP in neurones. However, ATP can be depleted under cerebral ischemia and hypoxia because the consumption of ATP continues while the synthesis of ATP is blocked due to the lack of O_2_ and glucose which are the substrates for ATP synthesis [14]. The energy supply of Na^+^/K^+^-ATPase will be reduced resulting in a decrease in enzyme activity. Decreased enzyme activity may also be caused by the unbalance of Na^+^ and K^+^. An increase in Na^+^ and water intracellularly can lead to cell edema [15].

In the present study, pathological changes in the cerebellum and brain stem were detected after sacrificing the SPV. The results indicated that after sacrificing the SPV, edema of cerebellar tissue occurred at 4 h, peaked at 8 h, continued to 12 h, and then reduced gradually. Brain stem edema occurred after sacrificing the SPV in the 4-h subgroup, while no obvious edema was found in the 8, 12, 24, 48, and 72 h groups. These results were consistent with some clinical observations.

Cerebral edema is an important factor that aggravates neurological dysfunction and it may cause serious complications after SPV sacrifice. Although the experimental animals did not show coma, death, and other serious complications, this may be due to limited sample size. More attention should be paid to the treatment of SPV. Cerebellar edema after the SPV were sacrificed may be due to the following reasons: (i) obstruction of the SPV caused poor circulation which can induce an increase in venous pressure. The increased venous pressure can drive free water to go through the capillaries and infiltrate the parenchyma and ventricle of brain; (ii) cerebrospinal fluid reflux may be obstructed and blood flow slowed down, resulting in ischemia and anoxia of the brain tissues leading to brain edema. At the same time, the increased venous pressure can lead to the gradual opening of the collateral circulation so that the venous reflux is reinstated. The fact that the edema caused by the sacrificing of SPV was alleviated gradually over time supports this theory. Based on the current study, the mechanism of brain stem and spinal cord edema is not very clear. However the mechanism may be similar to that of cerebellar edema.

In this experiment, brain stem edema is not obvious, however, there are three possible reasons for this phenomenon. First, the surrounding space of the brain stem is larger when compared with the cerebellum. Second, the brain stem does not have the clear dura mater boundary like the cerebellum. Third, the vascular compensation of brain stem region is relatively rich resulting in the brain stem having a good compensatory ability compared with the cerebellum.

The rabbit model used in the present study was very useful when studying the pathological changes of cerebellar and brain stem edema. Pathological changes can be observed over a wide range of time periods which can then be used to inform doctors of appropriate times to evaluate the condition of patients after sacrificing the SPV during surgical procedures.

In the present study, rabbits were used to establish the SPV sacrifice model. A previous study demonstrated that the distribution of blood vessels of rabbits was closer to primates compared with dogs and cats [16]. Observations from the current study found that the distribution of the blood vessels and the drainage area in the cranial posterior fossa of rabbit were highly similar to humans [10]. There are three main veins in the cranial posterior fossa, including the vein which drains the anterior region of the brain stem, the vein which drains the cerebellar petrosal surface, and the vein which drains the trigeminal nerve caudal. However, because of the species difference of the human and rabbit, some differences may exist between the human and rabbit such as a difference in number of branches of the veins and the number of main drainage veins of SPV, the SPV length, and the outer diameter of SPV. Although the cerebral edema in rabbits progressed rapidly and then recovered quickly postoperatively, differences anatomically and physiologically between rabbits and humans may not result in edema in humans. In this experimental model only healthy rabbits underwent the procedure whereas in patients undergoing SPV sacrifice may have various diseases. In operations to remove certain tumors, the effect of the SPV sacrifice seems to be more severe than in the treatment of trigeminal neuralgia, which was not shown in this experiment.

### Conclusion

The cerebellum and brain stem edema occurred after sacrificing the SPV. The cerebellum and brain stem edema can be observed at different time points using the rabbit model.

## Abbreviations

Na^+^/K^+^-ATPase: sodium/potassium-ATPase
SPV: superior petrosal vein
WC: water content
